# Virtual HDR CyberKnife SBRT for Localized Prostatic Carcinoma: 5-Year Disease-Free Survival and Toxicity Observations

**DOI:** 10.3389/fonc.2014.00321

**Published:** 2014-11-24

**Authors:** Donald Blake Fuller, John Naitoh, George Mardirossian

**Affiliations:** ^1^Division of Genesis Healthcare Partners Inc., CyberKnife Centers of San Diego Inc., San Diego, CA, USA; ^2^Division of Genesis Healthcare Partners Inc., Coast Urology Medical Group Inc., La Jolla, CA, USA

**Keywords:** CyberKnife, prostate cancer, dosimetry, HDR, brachytherapy, image guided, stereotactic body radiotherapy

## Abstract

**Purpose:** Prostate stereotactic body radiotherapy (SBRT) may substantially recapitulate the dose distribution of high-dose-rate (HDR) brachytherapy, representing an externally delivered “Virtual HDR” treatment method. Herein, we present 5-year outcomes from a cohort of consecutively treated virtual HDR SBRT prostate cancer patients.

**Methods:** Seventy-nine patients were treated from 2006 to 2009, 40 low-risk, and 39 intermediate-risk, under IRB-approved clinical trial, to 38 Gy in four fractions. The planning target volume (PTV) included prostate plus a 2-mm volume expansion in all directions, with selective use of a 5-mm prostate-to-PTV expansion and proximal seminal vesicle coverage in intermediate-risk patients, to better cover potential extraprostatic disease; rectal PTV margin reduced to zero in all cases. The prescription dose covered >95% of the PTV (V100 ≥95%), with a minimum 150% PTV dose escalation to create “HDR-like” PTV dose distribution.

**Results:** Median pre-SBRT PSA level of 5.6 ng/mL decreased to 0.05 ng/mL 5 years out and 0.02 ng/mL 6 years out. At least one PSA bounce was seen in 55 patients (70%) but only 3 of them subsequently relapsed, biochemical-relapse-free survival was 100 and 92% for low-risk and intermediate-risk patients, respectively, by ASTRO definition (98 and 92% by Phoenix definition). Local relapse did not occur, distant metastasis-free survival was 100 and 95% by risk-group, and disease-specific survival was 100%. Acute and late grade 2 GU toxicity incidence was 10 and 9%, respectively; with 6% late grade 3 GU toxicity. Acute urinary retention did not occur. Acute and late grade 2 GI toxicity was 0 and 1%, respectively, with no grade 3 or higher toxicity. Of patient’s potent pre-SBRT, 65% remained so at 5 years.

**Conclusion:** Virtual HDR prostate SBRT creates a very low PSA nadir, a high rate of 5-year disease-free survival and an acceptable toxicity incidence, with results closely resembling those reported post-HDR brachytherapy.

## Introduction

Stereotactic body radiotherapy (SBRT) is a potentially effective treatment method for clinically localized prostate cancer, radiobiologically well matched to prostate cancer due to its purported high sensitivity to fraction size, with encouraging short to intermediate-term SBRT results reported from a number of authors and institutions (1–7). Due to the still relative newness of the SBRT approach additional confirmatory data are desirable. If SBRT is ultimately demonstrated to have comparable efficacy and safety relative to other radiotherapy methods for prostate cancer, then several practical advantages could make a compelling case for its much more widespread adoption, including a very compressed treatment schedule versus conventionally fractionated radiotherapy, lower cost of delivery due to the much smaller number of treatments, and less invasiveness versus brachytherapy (8, 9).

Prior to the advent of SBRT, high-dose-rate (HDR) brachytherapy also demonstrated high efficacy and reasonable safety against localized prostate cancer (10–14). In addition to delivering favorable prostate cancer radiobiology due to its inherent large dose per fraction dosimetry (comparable to SBRT), HDR brachytherapy allows highly flexible radiation dose sculpting, with very conformal tumor volume coverage and increased dose in the peripheral zone of the prostate, so that the highest radiation dose matches the cancer cell distribution in this region (13, 15). The primary drawback of HDR brachytherapy is that it is an invasive procedure, requiring hospital admission, anesthesia, nursing support, and narcotic analgesia to place and manage the indwelling transperineal HDR catheters and deal with their attendant pain and risk of infection or thromboembolism.

CyberKnife^®^ (CK; Accuray Incorporated, Sunnyvale, CA, USA) SBRT is an image-guided device capable of delivering a quantitative radiation distribution to a precisely defined three-dimensional target volume, creating very sharp surrounding dose gradients, with sub-millimeter delivery accuracy contingent upon sufficient frequent intrafraction image-guidance frequency (16). In 2008, we described a dosimetry approach whereby SBRT could be designed to substantially recapitulate the intraprostatic and peri-prostatic isodose morphology of HDR brachytherapy, using CyberKnife^®^ device as the delivery system (16, 17). The obvious advantage of such an approach is the maintenance of HDR-like radiobiology, conformality and intraprostatic dosimetry control, while losing its invasive aspect. Although the main point of that initial manuscript was to demonstrate substantial equivalence in dose distribution between HDR prostate brachytherapy and “Virtual HDR” CyberKnife SBRT, the larger primary study endpoints were to evaluate the efficacy and toxicity of this specific treatment regimen. Sufficient time and patient accrual has now occurred, such that we now report disease-free survival (DFS) and toxicity for a larger number of consecutively treated patients at a single institution, at risk for a minimum of 5-years of post-treatment.

## Materials and Methods

Seventy-nine consecutive prostate cancer patients were treated with CK SBRT from July 2006 through July 2009, under one of two IRB-approved phase II prostate SBRT prostate monotherapy protocols, open to low-risk patients (DRE stage T1 – T2b, Gleason Score ≤6, prostate-specific antigen (PSA) level ≤10 ng/ml), and intermediate-risk patients (Gleason Score 7 or PSA level 10.1–20 ng/ml if other favorable characteristics still present) (18, 19). Our series included 40 low-risk and 39 intermediate-risk patients with a median presenting PSA level of 5.3 ng/ml (range 1.0–19.1 ng/ml). Fifty-one patients presented with Gleason score ≤6 disease, while the remaining 28 had Gleason score 7 disease (3 + 4 − 24 pts, 4 + 3 = 4 pts). Presenting patient characteristics are described in Table 1. All patients received 38 Gy in four fractions, a schedule that has been shown to be efficacious with HDR brachytherapy (20). Standard Kaplan Meier plots were used to describe biochemical-relapse-free survival outcomes by relapse definition (ASTRO, Phoenix).

**Table 1:** **Patient characteristics**.

Median age at tx (years)	68
Range	53–80
Risk group	No. of patients
Low	40
Intermediate	39
Gleason	No. of patients
5	1
6	50
7	28
Presenting PSA	No. of patients
<10 ng/ml	68
≥10 ng/ml	11
T-stage	No. of patients
1 = T1b	0
2 = T1c	48
3 = T2a	23
4 = T2b	8
Median prostate volume	39.8
Range	11.1–145.7
Presenting AUA score
Median	6
Range	0–29
Presenting SHIM score
Median	17
Range	1–25

### Treatment planning

**Figure 1:**
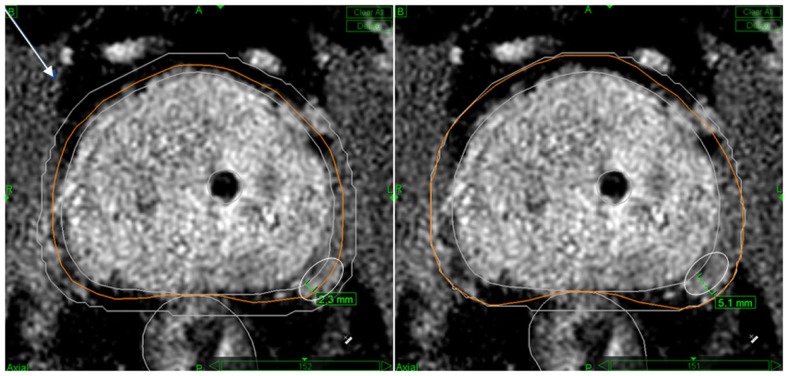
**Typical PTV margins used in this protocol for low-risk (left panel) and intermediate-risk (right panel) cases**. The protocol calls for Prostate + 2 mm CTV to PTV expansion margins (low-risk disease) and prostate + 5 mm unilaterally or bilaterally if Gleason 7 disease or PSA >10 ng/mL is present (intermediate-risk disease). Note “shaving” of the GTV to PTV margin to zero against the rectum in the midline for both risk-groups. Also note that a 2-mm PTV expansion tends to “split” the NVB (delineated by white ovoid contour) while a 5-mm PTV expansion tends to fully encompass it.

The planning target volume (PTV) for all cases included the prostate as defined by our prostate MRI imaging protocol, three-dimensionally co-registered with prostate CT imaging, matching fiducial to fiducial, and a 2-mm volume expansion in all directions, except posteriorly where the prostate abutted the rectum, where the margin expansion was reduced to zero based on reports that prostate cancer does not invade posteriorly in the midline beyond Denonvilliers’ fascia (21). Intermediate-risk patients had a 5-mm dorsolateral prostate-to-PTV expansion (limited to the side of the prostate harboring the intermediate-risk lesion) and ≥1 cm of contiguous seminal vesicle inclusion in the PTV to more effectively cover their increased risk and potential distance of extracapsular extension near the neurovascular bundle (NVB) (22). Typically, the 2-mm margin expansion used in favorable prognosis patients split the NVB as defined on T1-weighted, gadolinium-enhanced MRI, while the 5-mm expansion used for intermediate-prognosis patients fully encompassed it (Figure 1). The urethra was identified by insertion of a Foley catheter, which also provided another reference structure to use in MRI-to-CT image co-registration.

**Figure 2:**
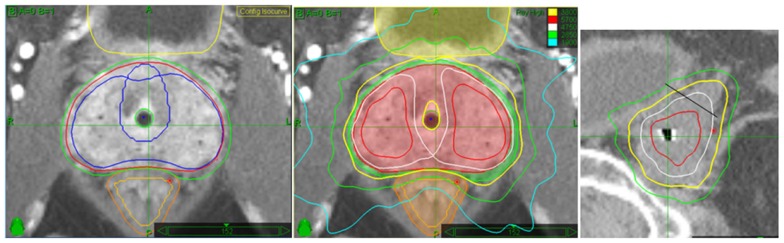
**This is an example of an intermediate-risk case with L-sided Gleason score 7 disease present**. Note the asymmetric GTV to PTV expansion margins (L Panel) (2 mm around the R lobe versus 5 mm around the L lobe) to more widely encompass the possibility of significant extracapsular extension on the side with the higher Gleason score. This creates wider prescription (yellow line) isodose coverage on the higher-risk side, and also tends to create larger volumes of dose escalation within the prostate on the more heavily involved side (center panel). Finally, note full prescription isodose coverage (yellow line) encompassing at least 1 cm of adjacent seminal vesicle on the sagittal image, required for intermediate-risk cases only (SV/prostate junction denoted by the oblique black line)(R panel).

Our CK SBRT treatment plans had a specific set of objectives and constraints, including a requirement of a minimum PTV prescription dose coverage of 95% (V100 ≥95%), a maximum PTV dose of 200% of the prescription dose (76 Gy), with greater than 200% classified as a minor protocol deviation only. Also required were a maximum rectal wall dose of 100% of the prescription dose (38 Gy), a maximum rectal mucosa dose of 75% of the prescription dose (28.5 Gy), a maximum urethra dose of 120% of the prescription dose (45.6 Gy), and a maximum bladder dose of 120% of the prescription dose (45.6 Gy). The rectal mucosa was defined as a solid structure formed by a 3-mm contraction of the rectal wall. The normal tissue dose limitation objectives were designed to resemble those commonly prescribed in the application of HDR brachytherapy (23). Typical Virtual HDR CyberKnife SBRT prostate treatment plans are demonstrated in Figure 2.

## Results

### Clinical outcomes

Of the 79 treated patients, all of whom were at risk for greater than 5 years, 6 were censored due to relapse or death from other causes, 20 were lost to follow-up while being disease-free between 6 and 54 months post-treatment (median – 42 months), and the remaining 53 disease-free patients were followed at least 5 years.

#### PSA response

**Figure 3:**
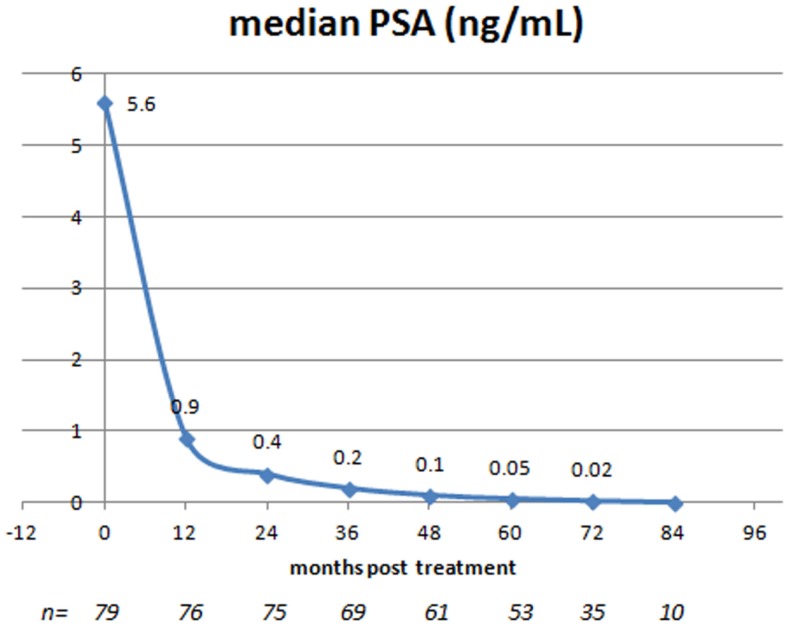
**Entire study population: median PSA at presentation and at annual follow-up intervals post-SBRT**.

The median baseline pre-treatment PSA level of 5.6 ng/mL decreased each year post-treatment, reaching a median value of 0.05 ng/mL at 5 years and thereafter decreasing further, to reach a nadir value of 0.02 ng/mL at 6 years post-treatment. The PSA-response to treatment at annual intervals is illustrated in Figure 3. Although the median PSA level for the entire treated population decreased with each annual follow-up interval out to 6 years, the phenomenon known as “benign PSA bounce” was also common and, in fact, only 24/79 (30%) patients had a continuous PSA decrease throughout their entire follow-up period. The remaining 55 patients (70%) had at least one sequentially increased PSA value during their follow-up evaluation, typically of small magnitude with subsequent resumption of a declining trend (median magnitude 0.2 ng/mL; maximum 3.3 ng/mL; median time 24 months out). Only 3/55 patients (5%) with a sequential PSA rise actually went on to develop biochemical disease relapse.

#### Disease-free survival

**Figure 4:**
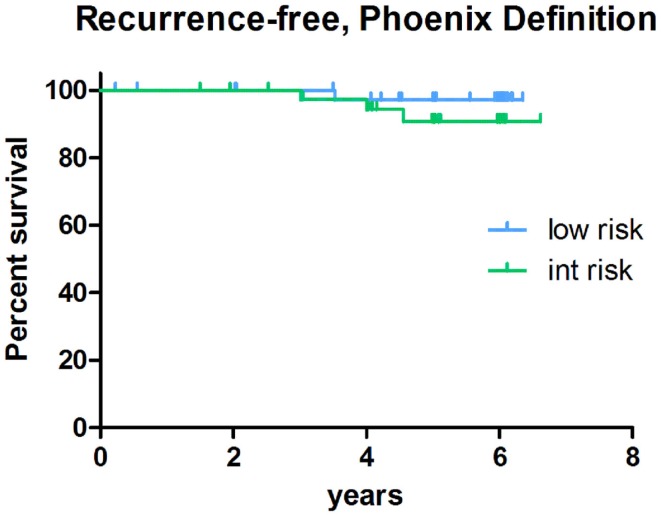
**Biochemical-relapse-free survival post-SBRT stratified by risk-group: Phoenix definition**.

**Figure 5:**
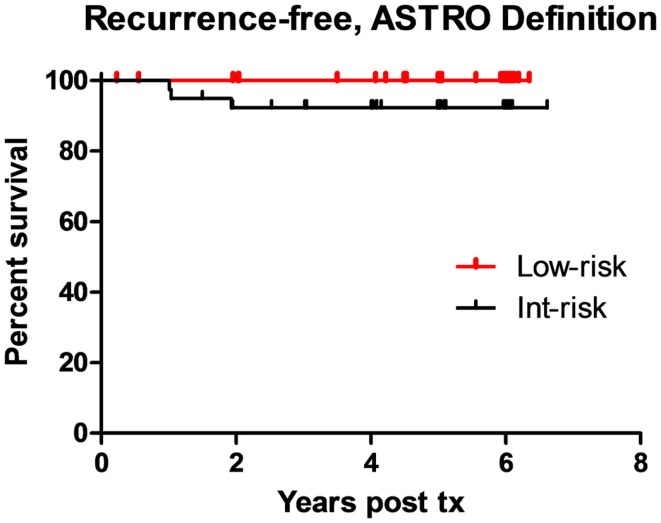
**Biochemical-relapse-free survival post-SBRT stratified by risk-group: ASTRO definition**.

With a minimum follow-up of 5-years, the biochemical-relapse survival rates measure 100 and 92% for low-risk and intermediate-risk cases, respectively, using the ASTRO biochemical-relapse-free definition. Corresponding low-risk and intermediate-risk DFS rates measure 98 and 92%, respectively, using the Phoenix definition. Of added note, the only low-risk patient classified as biochemically relapsed by Phoenix criteria, in fact, subsequently proved to have a benign PSA bounce rather than a true relapse. Biochemical DFS outcomes by risk group are illustrated in Figures 4 and 5. Clinically, the local relapse-free rate measured 100% for low-risk and intermediate-risk cases, while the distant metastasis-free survival rates measured 100 and 95% for low-risk and intermediate-risk cases, respectively. For the entire study population, the overall survival rate was 98%, with two deaths from intercurrent disease, and prostate cancer-specific survival of 100%.

#### Toxicity

**Figure 6:**
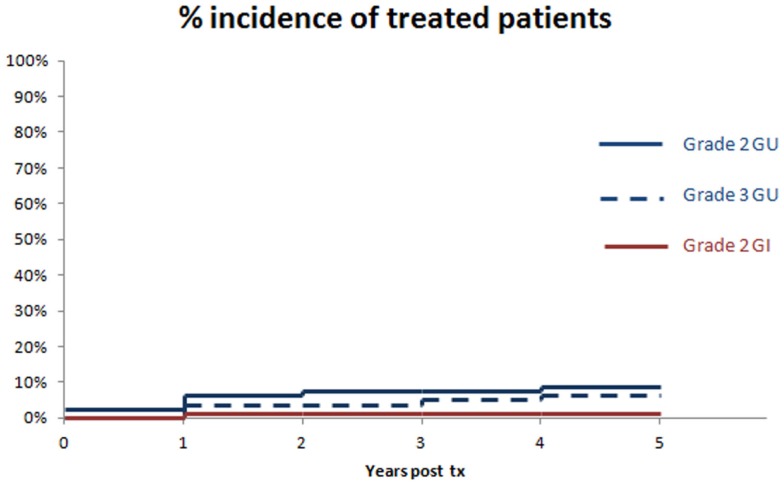
**Cumulative incidence of long-term grade 2 or higher GU or GI morbidity, annualized to 5 years**. There were no further events beyond 5 years in this study.

Toxicity scoring was by CTCAE v 3.0 criteria. The primary toxicity of this regimen for both acute and chronic time frames has occurred in the GU Domain, with 10 and 9% acute and chronic grade 2 toxicity rates, and 0 and 6% acute and chronic grade 3 GU toxicity rates, respectively. There were no instances of acute catheter-dependent urinary retention in this series. Acute and chronic toxicity in the GI domain has been far lower, with respective values of 0 and 1% for acute and chronic grade 2 or higher GI toxicity, respectively. There were no acute or chronic grade 3 or higher GI toxicity events. The actuarial rate of Grade 2 or higher GU and GI toxicity to 5 years is summarized in Figure 6.

**Figure 7:**
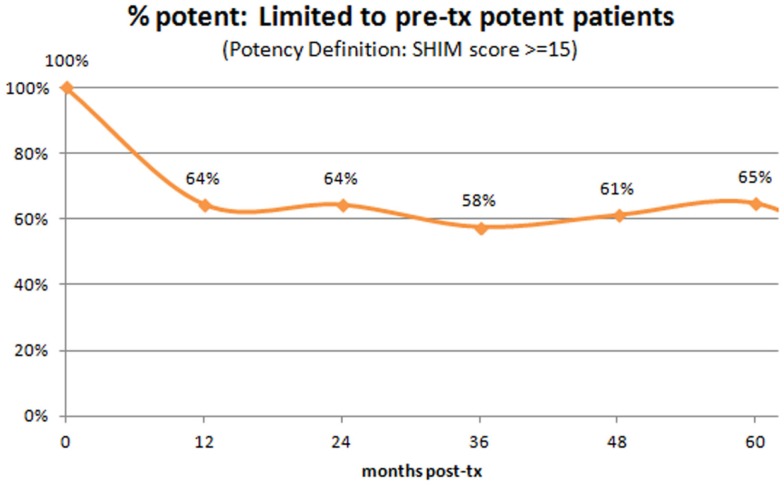
**Percent of patients with preserved potency – analysis limited to those who were potent pre-SBRT**. Note that virtually the entire loss incidence occurs within year one post-SBRT, with relatively stable rates thereafter.

We also evaluated potency at baseline and at annual intervals post-treatment (Figure 7). Of the entire study population, only 59% were sexually potent pre-treatment (defined as having a SHIM score of ≥15). Limiting the analysis to those patients who were potent pre-treatment, 65% remained potent 5 years out. Of note, virtually the entire loss appears to have occurred during year one post-SBRT, with a relatively stable rate of potency preservation thereafter to 5 years.

## Discussion

Our initial hypothesis that the CyberKnife may be used to deliver HDR-like dosimetry to the prostate non-invasively was originally demonstrated by respective dosimetry comparisons as reported in 2008 (17). Now, 6 years later, we report 5-year biochemical and clinical outcome results that also resemble those of HDR and permanent source brachytherapy in a number of ways as detailed below.

### PSA kinetics post-treatment

The PSA nadir seen after “HDR-like” SBRT is very low and continues to decrease even beyond 5 years. By standard lab reporting, which typically defines the PSA level as non-detectable at a value of <0.1 ng/mL, that particular PSA nadir threshold was reached 5 years out. By ultrasensitive PSA analysis, which increases the PSA detection sensitivity to very low levels, a further decrease to a median PSA nadir of 0.02 ng/mL is seen 6 years out, suggesting eventual “total PSA ablation” in the majority of patients by this specific SBRT treatment method and dose fractionation schedule. Similarly, following HDR brachytherapy and permanent source brachytherapy, PSA nadirs of ≤0.1 ng/mL have also been reported (24). Finally, “non-HDR-like” SBRT may also create near ablation PSA levels post-treatment, though this has not been universally observed and some SBRT series have reported modestly higher median PSA nadir values. (1, 6, 25). Potentially, this reflects a PSA ablation dose response aspect to non-HDR-like SBRT regimens, with a minimum threshold dose of 35 Gy in five fractions or higher (prescribed to a volume rather than the ICRU reference point) to routinely achieve a PSA nadir value of 0.1 ng/mL or less. Somewhat higher PSA nadir values have been reported following conventional fractionation external beam radiotherapy, with a mature IMRT median PSA nadir result of 0.6 ng/mL reported by a large, experienced IMRT center (24). Thus, it appears likely that HDR-like SBRT, aggressively fractionated non-HDR-like SBRT and brachytherapy are relatively radiobiologically more potent, typically ablating both prostate cancer and normal prostate tissue. The slightly higher PSA nadir seen post-conventional fractionation radiotherapy suggests that at least some cells survive in the high-dose region long-term, though this still does not establish *de facto* prostate cancer disease control superiority to the PSA-ablative methods, as the trace higher PSA levels seen post-conventional radiotherapy could be reflective of small amounts of surviving normal prostate glands as opposed to surviving cancerous glands. Of concern, however, is the clear correlation of subsequent relapse with higher post-radiotherapeutic PSA nadir levels, suggesting the possibility that total glandular ablation may indeed be required to maximize the log-term DFS probability (26–28). There was a large non-randomized series with mature follow-up that did indeed observe a significantly lower PSA nadir with brachytherapy versus IMRT, subsequently correlating this “ablation level” post-brachytherapy PSA nadir with eventual biochemical-relapse-free survival superiority, though no difference in the clinical DFS rate was observed (24). A higher rate of grade 2 GU and GI toxicity was also seen in the brachytherapy patients in this series, suggesting a greater biologic potency to adjacent normal tissues as well. Only well-controlled randomized clinical trials with very long follow-up would be able to assess DFS or toxicity differences definitively, unfortunately, and none are forthcoming soon.

### Disease-free survival

Our observed 100 and 92% 5-year biochemical-relapse-free survival rates for low-risk and intermediate-risk cases, respectively, with a 100% 5-year local relapse-free survival rate, appears to further corroborate the effectiveness and radiobiologic potency of this HDR-like SBRT treatment method and dose fractionation schedule. The few observed biochemical relapses in the intermediate-risk group with subsequent clinical relapse have been due to metastatic disease and these relapses have typically been seen within the first 3 years post-treatment. This suggests that occult disease beyond the prostate bed was present in these cases even before their diagnosis and treatment. Although 10-year data with a larger patient population are desirable to establish long-term efficacy, the fact that an “ablation” median PSA nadir level was obtained at a minimum post-SBRT follow-up interval of 5-years suggests that the DFS result will be durable and competitive with any other local prostate cancer treatment method described to date.

### Complications

Clearly, any radiotherapy regimen potent enough to produce a PSA nadir level approaching zero has toxicity potential, and we have indeed seen instances of grade 2 or higher toxicity in this series. The primary toxicity occurred in the GU domain. Acutely (<90 days), there was a 10% incidence of grade 2 GU toxicity and a 0% incidence of grade 2 GI toxicity. Acute GU toxicity typically peaked within the first month post-treatment, with an approximate 10 point median increase in the I-PSS score over baseline at 2 weeks out, with steady improvement thereafter. Although this specific SBRT regimen has substantially identical radiobiologic potency versus HDR brachytherapy, upon which our dose fractionation regimen was designed (3,800 cGy in four fractions), there have been no observed cases of acute urinary retention. Post-brachytherapy acute urinary retention has been reported following both permanent source and HDR prostate brachytherapy, with an incidence of 3–12% with evidence of a reduced incidence with technical refinements and increased experience of the implanting team in one of these reports (10, 29–31). Presumably, the major contributor to acute post-brachytherapy urinary retention is needle trauma. An obvious advantage of the current SBRT approach is the absence of invasive transperineal needle punctures or transcatheter delivery, and this advantage is clinically reflected by the total lack of acute post-SBRT catheter-dependent urinary retention. The insertion of a urethral catheter as practiced for “HDR-like” SBRT treatment planning to define the position of the urethra could also potentially contribute to very short-term GU toxicity (rare cases of grade 1 only in this series – no obstruction). Late toxicity is more likely a pure radiotherapy issue, with all of our grade 3 GU toxicity cases occurring beyond a year – long after full resolution of any short-term catheter-related GU toxicity issue.

Chronically (>90 days), we observed 9 and 6% rates of grade 2 and 3 GU toxicity, respectively. As the predicate series upon, which we based our Virtual HDR SBRT regimen was the HDR brachytherapy regimen described by the William Beaumont group in 2004, it is not surprising that the incidence of grade 3 GU toxicity in that series was comparable to our own observation (8% with HDR brachytherapy in that series vs. 6% with “HDR-like” SBRT in the current series) (12). Our prescribed dose is identical to their HDR dose prescription, and delivered to a substantially equivalent target volume. Although brachytherapy-associated catheter trauma may contribute to short-term toxicity (<90 days), we presume that remaining or worsening late toxicity is more clearly a reflection of the radiation effect itself; nearly identical chronic grade 3 toxicity profiles between our series and its predicate HDR brachytherapy series would appear to validate this presumption (12). Regardless of the methodology used to deliver this dose fractionation schedule, the best way to reduce the incidence of serious chronic urinary tract toxicity probably lies in the patient selection process. Relative caution in treating patients with preexisting urologic issues such as serious, chronic obstructive uropathy would most likely reduce the incidence of serious chronic GU toxicity, regardless of the specific delivery methodology. If “HDR-like” SBRT is practiced in the absence of accurate urethra localization, the incidence of late grade 3 GU toxicity could potentially be further increased, due to variable urethral location from patient to patient that could create intended superimposition of extreme intraprostatic radiotherapy dose escalation over some or all of an incorrectly “estimated” urethra location. Finally, urologic instrumentation post-SBRT may also contribute to serious late GU toxicity. At least one of our cases of late grade 3 GU toxicity were immediately preceded by a cystoscopy procedure and a similar instrumentation versus grade 3 GU complication correlation was previously reported by King et al. (32).

### Potency preservation

Only 59% of our patients were sexually potent pre-SBRT, and even that may be an optimistic assessment, as we defined “sexually potent” in this series as having a SHIM score of ≥15, whereas “perfect” sexual function is defined by a SHIM score of 25. Some degree of preexisting sexual dysfunction will inevitably be present in any prostate cancer series and ours is no different, as their median age was 68 years. Of patients who met our definition of sexually potency prior to SBRT, 65% retained sexual potency 5 years post-treatment, with virtually the entire potency loss incidence seen within the first year post-SBRT and relatively stable potency preservation rates seen thereafter. Undoubtedly, in addition to the effect of radiotherapy itself on the NVB and/or penile bulb, other factors may strongly influence the long-term potency outcome, including patient age, presence versus absence of preexisting mild ED, vascular insults such as diabetes, hypertension, and tobacco-induced small vessel disease. This series is too small to effectively evaluate subgroups, though we expect our larger companion, multi-institutional “Emulating HDR” prostate SBRT series to shed further light on this issue (33). In fact, there has been a preliminary potency outcome correlation reported in that series, with advancing age and preexisting decreased SHIM score both strongly predictive of an increased rate of subsequent post-SBRT potency loss (33).

### Summary

We initially demonstrated that CyberKnife robotic SBRT represented a non-invasive method to deliver radiation dose distributions that very closely resembled those delivered by HDR brachytherapy (17). We now have 5-year clinical outcomes that demonstrate a similar DFS rate and PSA nadir versus that seen with HDR prostate brachytherapy, minus the short-term hospitalization, catheter trauma, and acute urinary retention potential. Grade 2 or higher long-term GU toxicity is seen with a similar incidence between HDR-like SBRT versus actual HDR brachytherapy, with stricter patient selection or reduced SBRT dose intensity representing potential strategies to reduce this. HDR-like SBRT appears to represent a biologically potent local prostate cancer treatment method, seemingly effectively combining “external beam-like” non-invasiveness with “brachytherapy-like” convenience and biologic potency. As such, this method appears to be a potentially valuable addition to the prostate cancer local treatment armamentarium.

## Conflict of Interest Statement

The authors declare that the research was conducted in the absence of any commercial or financial relationships that could be construed as a potential conflict of interest.

## Abbreviations

CK, CyberKnife; CT, computed tomography; HDR, high-dose rate; IGRT, image-guided radiotherapy; IMRT, intensity-modulated radiotherapy; IPSS, international prostate symptom score; MRI, magnetic resonance imaging; PSA, prostate-specific antigen; PTV, planning target volume; SBRT, stereotactic body radiotherapy; US, ultrasound.
